# A glossy mutant in onion (*Allium cepa* L.) shows decreased expression of wax biosynthesis genes

**DOI:** 10.3389/fpls.2023.1245308

**Published:** 2023-08-23

**Authors:** Tushar Kashinath Manape, Parakkattu S. Soumia, Yogesh P. Khade, Viswanathan Satheesh, Sivalingam Anandhan

**Affiliations:** ^1^ Crop Improvement Section, Indian Council of Agricultural Research (ICAR)-Directorate of Onion and Garlic Research, Pune, Maharashtra, India; ^2^ Genome Informatics Facility, Office of Biotechnology, Iowa State University, Ames, IA, United States

**Keywords:** cuticular wax, glossy, mutant, wax biosynthesis, RNA-seq

## Abstract

Cuticular wax is a characteristic feature of land plants that provides protection against both biotic and abiotic stresses. In this study, a glossy mutant lacking an epicuticular wax layer was identified in the γ-irradiated M_2_ mutant population of the onion cultivar Bhima Super. The inheritance of the mutant’s glossy phenotype was determined to be recessive and single locus. Scanning electron microscopy analysis showed poor accumulation of wax crystals in the glossy mutant, concentrated near the stomata. The plant height, number of leaves per plant, and stomatal parameters of the mutant were similar to the wild-type. RNA-seq was used to comprehend the expression variations of waxy cuticle-related genes in the glossy mutant and its wild-type waxy cultivars. Differential gene expression analysis of the RNA-seq data revealed that the genes involved in wax biosynthesis, such as *AcCER1, AcCER26, AcMAH1*, and *AcWSD1*, were downregulated by 2.72, 1.74, 2.59 and 2.12-fold, respectively, in the glossy mutant respectively. The expression patterns of these four unigenes were validated using semi-quantitative RT-PCR. The glossy mutant displayed a substantial 3.5-fold reduction in cuticular wax load compared to the wild-type due to the significant downregulation of these wax biosynthesis genes. These findings represent early advancements in understanding the molecular mechanisms of wax biosynthesis in onions. Furthermore, they provide a foundation for utilizing the glossy mutant trait in breeding programmes to enhance stress and pest resilience.

## Introduction

Onion is an important vegetable crop and is preferred for its flavor profile. India is the second-largest contributor to onion production in the world, with a substantial output of 26,738,000 metric tons. Moreover, India ranks among the top consumers and exporters of onions on a global scale (FAOSTAT, 2021). The leaves of onions display characteristic white wax deposits on their surfaces.

Cuticular wax is an important adaptation of terrestrial plants, forming a protective outer layer on plant surfaces (Edwards et al., 1996; Yeats and Rose, 2013). This layer helps to maintain the water balance of plants by regulating non-stomatal water loss and provides a barrier against a range of biotic and abiotic stresses, including drought, cold, UV radiation, pests and pathogens. The composition of cuticular wax varies between species, as well as within a species between different organs, developmental stages and tissue types (Yeats and Rose, 2013). In addition, environmental factors such as light, drought, humidity temperature can also influence the distribution of cuticular wax within a species. The distribution and chemical composition of cuticular wax can affect a plant’s ability to tolerate biotic and abiotic stress (Yeats and Rose, 2013; Serrano et al., 2014; Singh et al., 2018), making it an important trait for breeders to consider when developing crops with improved stress tolerance. The crystal structure of cuticular wax also varies based on its composition and can impact the survival, feeding and oviposition of insects. The wax composition or wax load on the leaf surface has been shown to be important in determining plant tolerance to thrips in onion. Leaf wax composition analysis shows that Hentriacontanone-16 is a major component of cuticular wax that determines the total wax load in leek and onion leaves (Rhee et al., 1998; Munaiz et al., 2020). The wax related phenotype is mainly determined by the quantitative variation in accumulation of hentriacontanone-16 in visually different phenotypes of onion such as waxy, semi-glossy and glossy (Munaiz et al., 2020). Genetic analysis of naturally occurring mutants exhibiting a glossy phenotype in BianGan Welsh onion (Yang et al., 2017) and in the onion varieties ‘Australian Brown’ and ‘White Persian’ (Jones et al., 1944; Munaiz and Havey, 2020) has shown that this trait is controlled by a recessive allele and is determined by different loci.

Cuticular wax is composed of a complex mixture of very long chain of fatty acids (VLCFAs) and their derivatives, such as aldehydes, alkanes, ketones, fatty alcohol and even cyclic compounds (Yeats and Rose, 2013). The fatty acids are synthesized in the chloroplast and exported to the endoplasmic reticulum for elongation (Block and Jouhet, 2015). Further, the cuticular wax are synthesized in epidermal cells and deposited on the surface. The biosynthesis of cuticular wax is a complex process that involves the coordination of several genes. These cuticular wax biosynthesis genes have been identified in several plants, including maize, rapeseed, tomato and model plants such as *Arabidopsis* and rice. *Eceriferum 6 (CER6), Eceriferum 10 (CER10), Glossy 8A (GL8A), Glossy 8B (GL8B), Formate dehydrogenase (FDH), Fatty Acid Elongation1 (FAE1), 3-ketoacyl-CoA synthase1 (KCS1) and Protein-tyrosine phosphatase 2 (PAS2)* genes then elongate these C16 and C18 CoA esters to form VLCFAs precursors mediated through long-chain acyl-CoA synthetase (LACS) enzymes. KCS, KCR, HACD, and CER26, which are endoplasmic reticulum-associated fatty acid elongases, work sequentially to synthesize fully saturated long chains acyl-CoA. These acyl-CoAs then enter either the decarbonylation pathway or the acyl reduction pathway. The decarbonylation pathway involves the conversion of long chain fatty acyl-CoAs into aldehydes, alkanes, secondary alcohols and ketones through the action of *FAR, CER1, CER4*, and *MAH1* genes. The acyl reduction pathway, which includes *FAR, CER4*, and *WSD1* genes, converts fully saturated long chain acyl-CoAs into primary alcohols and wax esters. These waxes are transported to plasma membrane by an unknown mechanism and subsequently into the cell wall by ABC transporters (*CER5* and *ABCG*). Further, transport of wax compounds across the cell wall is facilitated by lipid transfer proteins to the final destination at the cuticle (Li-Beisson et al., 2013).

In this study, we report a glossy mutant in onion developed through γ-irradiation mutagenesis. A comparative transcriptome analysis between the glossy mutant and its wild-type counterpart revealed significant differences in the wax biosynthesis pathway genes between the glossy mutant and the wild-type. These observations highlight the importance of comprehending the molecular mechanisms involved in wax biosynthesis in onions, as it holds great potential for enhancing crop quality and resilience to stress.

## Materials and methods

### Plant material

The present study was conducted during 2019-2022 at the Indian Council of Agricultural Research - Directorate of Onion and Garlic Research (ICAR-DOGR), situated at the latitude (27°19’00.2 N) and longitude (82°25’00.1E) Pune, Maharashtra, India 553.8 meters above sea level. Onion being a biennial crop, its seed production was done during *rabi* season and the subsequent generations were grown in *kharif* during 2019-2021. The glossy mutant phenotype was observed in the seed-germinated M_2_ population of γ-irradiation-treated Indian short-day onion cv. Bhima Super (B. Super) mutant line (IR-300-53). Bulbs of the M_2_ generation γ-irradiated onion (glossy type) and its non-mutagenized counterpart [wild-type (WT)/waxy], were planted in a greenhouse under a 16/8-h light/dark photoperiod at 25°C. The phenotypic data, including plant height and number of leaves per plant were recorded at 75 days after transplanting (DAT). The glossy mutants were both selfed and outcrossed with the wild-type. Seeds from the subsequent F_1_ and M_3_ generations were sown in the greenhouse. The F_1_ plants were backcrossed with the glossy mutant and the number of glossy and waxy phenotypes were recorded in the M_3_, F_1_ and BC_1_F_1_ generations. All plant studies were carried out in accordance with relevant institutional, national, and international guidelines and legislation.

### Scanning electron microscopy

Onion leaves are isobilateral, having equal stomatal density on both sides of the leaf. The third leaf from the center of both the WT waxy and M_3_ mutant glossy plants were torn into 3×3 mm^2^ pieces using tweezers and mounted on aluminium stubs. The leaf specimens were then sputter-coated with gold and examined for the micro-structure analysis using an Environmental Scanning Electron Microscope (ESEM) (FEI, Quanta 200 and ESEM Mode) at Sophisticated Analytical Instrument Facility (SAIF) of Indian Institute of technology (Bombay, India). Electron micrographs of leaf surfaces from three replicate plants were taken for each line and used to visually estimate the shape of the epicuticular waxes and cuticular striation. The stomatal count was measured using the IMAGEJ software v. 1.43u. Stomatal index was calculated using the formula, Stomatal index (%) = (S/S+E) × 100; where, S and E are the number of stomata and epidermal cells, respectively; in the microscopic field of view (FOV).

### Epicuticular wax coverage

The epicuticular wax content was extracted using the colorimetric method as described by Ebercon et al. (1977). Intact onion leaves were immersed in 15 ml distilled chloroform for 10 s and kept in a boiling water bath until the smell of chloroform was no longer detected. A 5 ml wax reagent (K_2_Cr_2_O_7_ prepared in conc. H_2_SO_4_) was added to each tube and kept at 60°C in a water bath for 30 min. After cooling, 12 ml of deionized water was added to each tube, and the solution was filtered through a filter paper. The absorbance was measured at a wavelength of 590 nm using SPECTROstar Nano microplate reader. The wax content was calculated using standard Polyethylene Glycol-3000 and expressed as mg per cm^2^.

### RNA sequencing, *de novo* assembly and sequence clustering

Total RNA was isolated from 6-weeks-old leaves of wild-type and M_3_ generation glossy mutant plants in 2 biological replicates using TRIzol reagent. The mRNA was then isolated and subjected to quality control (QC) before being used for paired-end (PE) library preparation. The libraries were sequenced on the Illumina NovaSeq 6000 platform using 2×150 bp chemistry at Eurofins India Pvt. Ltd. (Bangalore, India). The generated raw sequence datasets were deposited in the NCBI in the Short Read Archive (SRA) database with Bioproject accession number PRJNA943431. The sequenced raw paired-end reads of all samples were processed to obtain clean and high-quality concordant reads using Trimmomatic v0.38 (Bolger et al., 2014) to remove adapters, ambiguous reads (reads with unknown nucleotides “N” larger than 5%), and low-quality sequences (reads with more than 10% quality threshold (QV) < 20 phred score. The resulting high-quality (QV>20) paired-end reads were used for *de novo* assembly using Trinity v2.8.4 (Henschel et al., 2012) with a kmer of 25, and the mapped high quality reads of all samples were pooled together for assembly into transcripts.

### CDS prediction, functional annotation and gene ontology analysis

The assembled transcripts were then further clustered together using CD-HIT-EST-4.6 to get unigenes. Only those unigenes which were found to have >90% coverage at 3X read depth were considered for downstream analysis. To identify sample-wise CDS from the pooled set of CDS, reads from each of the samples were mapped onto the final set of pooled CDS sequences using Bowtie mapper v2.4.4 (Langmead and Salzberg, 2012). TransDecoder v5.3.0 was used to predict coding sequences from the unigenes. Functional annotation of the CDS was performed using the DIAMOND program (Buchfink et al., 2015) with BLASTX alignment mode to find the homologous sequences for the genes against non-redundant protein database (NR) from NCBI. Gene ontology (GO) analyses of identified CDS of each sample were carried out using Blast2GO program (Conesa et al., 2005). GO assignments were used to classify the functions of the predicted CDS. GO mapping was used to group identified genes into three main domains: Biological Process (BP), Molecular Function (MF) and Cellular Component (CC). To identify the potential involvement of the predicted CDS in biological pathways, all the identified CDS for each of the samples were mapped to reference canonical pathways in KEGG (Eudicots database) using KEGG automated annotation server, KAAS (Moriya et al., 2007).

### Differentially expressed gene analysis

The unigenes were used as the transcriptome reference for quantifying transcript abundance using Salmon version 1.8.0. A reference transcriptome index was created using the salmon “index” command. Transcript quantification was performed using the salmon “quant” command. The read counts generated was used for differential expression analysis with DESeq2 version 1.36.0 (Love et al., 2014). Genes that with absolute log fold change > 1 and adjusted p-value < 0.05 were determined to be differentially expressed geneS (Love et al., 2014; Patro et al., 2017).

### Semi-quantitative RT-PCR analysis

Total RNA was isolated from 100 mg shoot tissues of M_3_ glossy mutant and wild-type B. Super plants (with 2 biological replicates of each) using RNeasy^®^ Plant Mini kit (Qiagen). The RNA was quantified using Nanodrop (UV/Vis Nano spectrophotometer, Nabi, Metler Toledo). Total RNA (1 µg) was treated with DNase I, RNase free kit (Thermo Scientific) to remove any remaining DNA. First-strand cDNA synthesis was prepared using SuperScript™ IV Reverse Transcriptase kit (Invitrogen) as per manufacturer’s instructions and quantified using Nanodrop. First-strand cDNA (30 ng) was used for semi-quantitative RT-PCR analysis to validate the differentially expressed genes involved in the wax-biosynthesis pathway. Gene-specific primer sets were used, and the PCR reactions were performed according to the conditions described in **Table S1**. PCR products were run on 3% agarose gel and the image was captured with a digital camera under a UV light (Vilber Lourmat, ECX20.W). The band area was calculated using the ImageJ analyser tool and the band area of the wild-type was compared with the glossy mutant in terms of log_2_ fold change.

### Statistical analysis

To evaluate the variation in physical parameters between the glossy mutant and wild-type (WT) plants, a one-way analysis of variance (ANOVA) was employed using Statistical Analysis Software, Version 9.2 (Sas Institute, 2009). Mean comparisons were performed using the least significant difference (LSD) approach at a significance level of P=0.05. Chi-square analysis was performed in the segregating populations to understand the segregation pattern of the glossy mutant.

## Results

### Identification, phenotypic variance and segregation of glossy mutants

Four glossy green mutant phenotypes were observed in the M_2_ population of γ-irradiation-treated onion mutant line (B. Super-IR-300-53) (**Figure S1**). Bulbs from the M_2_ glossy mutant phenotype were harvested and planted separately under greenhouse conditions. The average plant height was 49.5 ± 2.2 cm and 57.5 ± 4.8 cm, and the number of leaves per plant was 6.5 ± 0.6 and 6.75 + 0.9 in M_2_ glossy mutant and WT; respectively. No significant differences were found between the glossy mutant and wild-type plants, with respect to plant height and number of leaves per plant. M_2_ plants were selfed and the subsequent M_3_ generation was found to be glossy. F_1_ plants derived from the cross between M_2_ and WT exhibited the waxy phenotype. However, when F_1_ plants were backcrossed with M_3_ glossy mutant, the subsequent BC_1_F_1_ population segregated in a 1:1 ratio (waxy: glossy).

### Leaf surface microstructure and morphology

**Figure 1:**
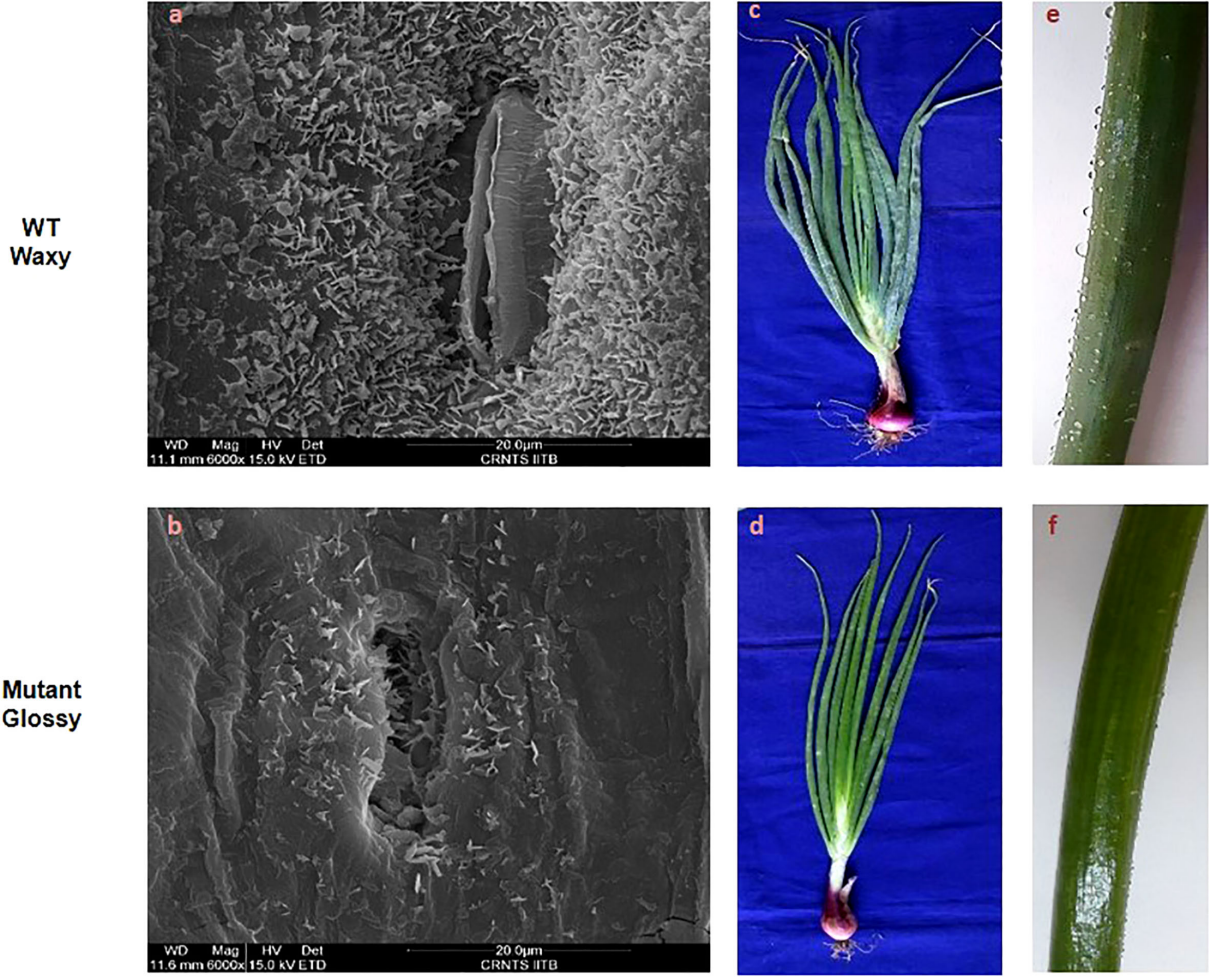
Leaf surface microstructure and morphology of WT and glossy mutant. Comparative scanning electron micrographs of leaf surface at magnification of 6000x of **(A)** WT waxy, **(B)** mutant glossy. Large quantities of spiky, needle-like crystals microscopic wax crystals were uniformly covered on the leaf surface of waxy WT onion, whereas in glossy type, relatively few wax crystals were scattered around the stomatal region alone. **(C)** Greenish gray (glaucous) phenotype of WT waxy, **(D)** Shiny glossy green phenotype of mutant glossy. **(E)** WT waxy onion leaf surface showing water droplets due to its hydrophobic nature, **(F)** water droplets were easily adhered and coalesced on to the leaf surface of mutant glossy.

**Figure 2:**
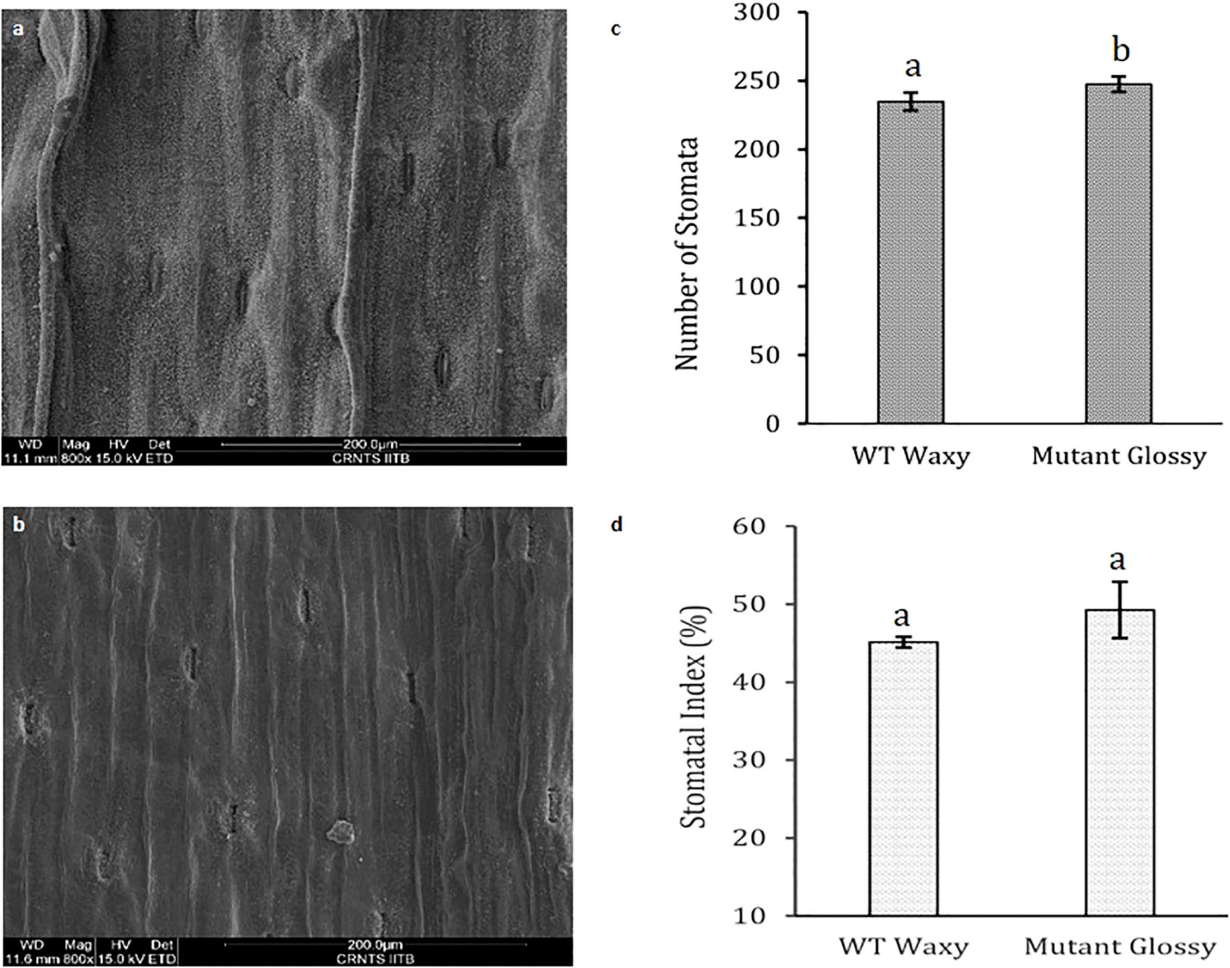
Leaf stomatal parameters in WT and glossy mutant. Comparative scanning electron micrographs of leaf surface at magnification of 800x of **(A)** WT waxy, **(B)** mutant glossy, **(C)** Number of stomata in 200 µm length in WT waxy and mutant glossy lines. **(D)** Stomatal index of WT waxy and mutant glossy lines. Stomatal index was calculated in percentage using formula, SI= (S/S+E) x 100 where, S and E are the number of stomata and epidermal cells respectively in 200 µm length microscopic view field. The bars indicate mean ± SE of 3 replicates. The lowercase letters "a and b" indicates the significant difference.

The bright green leaf of glossy mutant was in stark contrast to the glaucous leaf of WT in the experimental field. The color variation of the onion foliage is directly correlated with the amount and type of epicuticular wax deposits. SEM micrographs (**Figure 1A**) revealed large quantities of spiky, needle-like microscopic wax crystals uniformly covering the leaf surface of waxy WT onion, while in the glossy type, relatively few wax crystals were scattered around the stomatal region alone (**Figure 1B**). The bright green leaf of glossy mutant (**Figure 1D**) was distinguishable from the waxy leaf of the wild-type onion (**Figure 1C**). The foliage of the mutant onion appears shiny green, and water droplets easily form and adhere to leaf surfaces after the leaves are sprayed with water (**Figure 1F**). In contrast, the leaves of wild-type are greenish-grey, due to higher wax deposits (**Figure 1E**). The glossy leaves of mutant onion showed a wet phenotype (hydrophilic) as water droplets easily adhered onto the surface and coalesced. However, wild-type onion was more hydrophobic as water droplets formed beads due to a dense layer of epicuticular wax. The number of stomata and stomatal index were measured in the glossy mutant and wild-type onions. The results revealed a significant difference between the two genotypes: the mutant had 247 stomata with a stomatal index of 49, while the wild-type had 225 stomata with a stomatal index of 45 (**Figure 2**).

### RNA-seq assembly and analysis

RNA sequencing of 6-week-old leaves of wild-type onion (C1 and C2) and its glossy mutant (G1 and G2) was performed in duplicate using the Illumina platform (NovaSeq6000) with 2 × 150 bp chemistry. After stringent quality assessment and data filtering, a total of 104.33 million paired-end were generated, corresponding to 30.26 Gb of sequence data. Details of high-quality reads, transcripts generated, unigenes identified, predicted CDSs, functional annotation and classification of predicted CDSs, identification of differentially expressed genes (DEGs) and pathway enrichment analysis are summarized in **Supplement S1**. Before identification of DEGs, the annotated CDs from two biological replicates of each glossy M_3_ mutant and wild-type were pooled together independently and expression of unigenes were normalized. A total of 596 annotated unigenes were found to be differentially expressed between wild-type and glossy mutant, with 295 upregulated and 301 downregulated genes in the mutant (**Supplementary Sheet S2**).

### Profiling cuticular wax biosynthesis pathway genes

**Figure 3:**
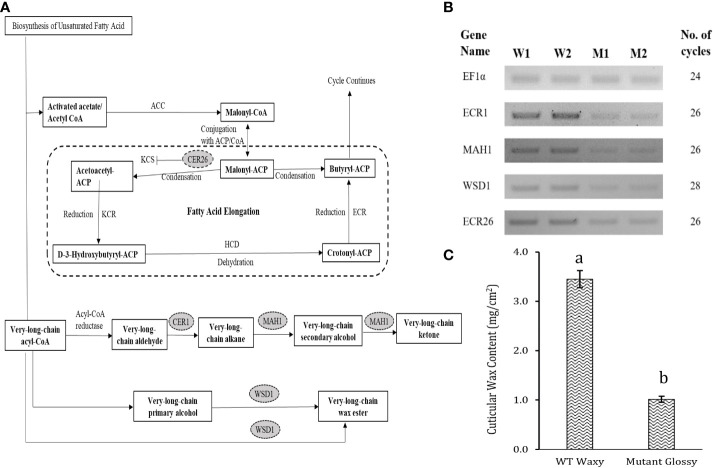
Downregulation of cuticular wax biosynthetic genes in the onion glossy mutant. **(A)** Basic cuticular wax biosynthetic pathway. **(B)** Oval highlighted genes were downregulated genes in glossy mutant onion lines by RNA-seq analysis. Question marks and dotted lines indicate unidentified enzymes and processes, respectively, **(B)** Validation of 4 downregulated genes of cuticular wax biosynthesis pathway by semi-quantitative RT-PCR. W1 and W2 indicates 2 biological replicates of WT waxy, whereas M1 and M2 indicates 2 biological replicates of mutant glossy. Total RNA was extracted from 6-week-old shoot tissues and the transcript levels were examined using semiquantitative RT-PCR. **(C)** Epicuticular wax content in WT waxy and mutant glossy lines. The bars indicate mean ± SE of 3 replicates. The lowercase letters "a and b" indicates the significant difference.

Out of 301 downregulated unigenes, 4 critical genes related to wax biosynthesis pathway *i.e. AcMAH1, AcWSD1, AcCER1* and *AcCER26* were significantly downregulated in the glossy mutant (**Figure 3A**). There was 1.74, 2.72, 2.12 and 2.59-fold decrease in the transcripts level of *ECR26, ECR1, WSD1* and *MAH1* genes, respectively (**Table 1**). To validate the RNA-seq results, expression levels of these genes were evaluated by semi-quantitative PCR, which also showed down-regulation of these genes in the glossy mutant (**Figure 3B**; **Figure S2**). Semi-quantitative PCR analysis had shown 1.17, 2.19, 1.61 and 1.95-fold down-regulation of *ECR26, ECR1, WSD1* and *MAH1* genes, respectively. The results of the semi-quantitative PCR were consistent with those of the RNA-seq analysis (**Table 1**).

**Table 1:** Differentially expressed unigenes associated with cuticular wax biosynthesis.

Sl. No.	Unigene ID	SwissProt annotation	COG class annotation	Log_2_ fold change			
				Semi-qRT PCR		RNA-seq	
				Waxy WT	Glossy mutant	Waxy WT	Glossy mutant
1	g304621	Protein ECERIFERUM 26	Lipid transport and metabolism	1	-1.17	1	-1.74
2	g367838	Protein ECERIFERUM 1	Lipid transport and metabolism	1	-2.19	1	-2.72
3	g50966	O-acyltransferase WSD1	Lipid transport and metabolism	1	-1.61	1	-2.12
4	g259833	alkane hydroxylase MAH1	Lipid transport and metabolism	1	-1.95	1	-2.59

## Discussion

### Segregation pattern of glossy mutant

The F_1_ plants derived from the cross between M_2_ and WT exhibited a waxy phenotype, revealing no segregation and thereby indicating the possibility of a recessive mutation. When F_1_ lines were back-crossed with M_3_ glossy mutant lines, BC_1_F_1_ lines showed an equal mixture of waxy and glossy mutants (**Table 2**), which confirmed the single-locus recessive gene inheritance of the glossy mutant phenotype. In consistent with our results, several studies had also shown that glossy mutant trait is controlled by single recessive gene/locus in maize (Bianchi et al., 1979), cabbage (Zeng et al., 2017; Liu et al., 2018), Welsh onion (Yang et al., 2017), Barely (Luan et al., 2017) and onion (Munaiz and Havey, 2020).

**Table 2:** Segregation pattern of glossy mutant lines in different populations.

Population	Total no. of plants	Phenotype	
		Waxy WT	Glossy mutant
M2	4	0	4
M3	15	0	15
F1	17	17	0
BC_1_F_1_	21	11	10^*^
WT	34	34	0

*Chi square analysis was performed in BC_1_F_1_ population. Calculated p value is 0.83. The difference was statistically non-significant.

### Epiculticular wax coverage and morphology of glossy mutant

The glossy mutant onion from the present study exhibited a distinct bright green phenotype compared to the WT waxy onion. The type and quantity of epicuticular wax deposits influences leaf color and leaf wettability. Damon and Havey, 2014, had visually classified onions as ‘‘glossy’’ or ‘‘semi-glossy,’’ on the basis of low to intermediate amounts of epicuticular waxes. Glossy mutant plants showed significantly fewer wax crystals, mainly concentrated around the stomatal region, giving it a greenish-grey color in contrast to WT. The shape of these wax crystals may vary based on self-assembly as sheet, filiform, tubular, and granular. According to descriptions of epicuticular wax crystals by Jeffree (1986), the spiky crystals on onion foliage are likely formed by a ketone. Studies by Gülz et al. (1992) and Gülz et al. (1993) concluded that characteristic lipid crystals can be observed on leaves if one lipid class comprises at least 40% of the total profile. Therefore, the spiky, needle-like microscopic wax crystals that predominate onion foliage can possibly be ketone(s). The leaves of the glossy mutant exhibit a hydrophilic property, facilitating the easy adhesion and coalescence of water droplets on their surface. In contrast, the wild-type onion has a dense layer of epicuticular wax, rendering it more hydrophobic and causing water droplets to form beads on its surface. These findings are consistent with previous studies conducted on rice and Welsh onion, highlighting the role of epicuticular wax in influencing leaf color, hydrophobicity, and water droplet behavior (Qin et al., 2011; Yang et al., 2017). Our results are in accordance with Yang et al. (2017), where the glossy mutant and wild-type of Welsh onion showed no significant differences in stomatal density.

### Cuticular wax biosynthesis genes

The cuticular wax biosynthesis and secretion pathway affects its content and composition. Studies on plant epicuticular wax biosynthesis have revealed ketones, including hentriacontanone-16, derived from the decarbonylation pathway involving aldehydes and alkanes as intermediates (Kunst and Samuels, 2003). Previous studies have provided a strong evidence that hentriacontanone-16 is the predominant epicuticular wax on onion (Damon et al., 2014) and leek (Rhee et al., 1998) foliage. The proteins MAH1 and CER1 are involved in the decarbonylation pathway, where CER1 converts long chain aldehydes to alkanes and MAH1 converts alkanes into secondary alcohols and ketones (Aarts et al., 1995; Greer et al., 2007). According to Jeffree (1986), the spikey wax crystals deposited on onion foliage is likely to be ketone which is in accordance with our results.

Mutations in the *cer1* gene result in glossy green stems with decreased wax deposition in *Arabidopsis*, while mutations in the *mah1* gene lead to glaucous inflorescence stems without affecting epicuticular wax crystals (Aarts et al., 1995; Greer et al., 2007). WSD1 participates in the acyl reduction pathway, and involved in synthesis of wax esters, a component of wax. *Arabidopsis wsd1* mutant had significantly reduced wax ester levels in the stem wax (Li et al., 2008). Leaf surface wax biosynthesis was broadly affected in glossy mutant of *B. napus* due to suppression of *CER1* and other wax-related genes (*MAH1, WSD1, CAC3, FATB, ABCG28*, *etc*.). Previous research has shown that upregulation of *CER1*, *WSD1* and *MAH1* genes triggered biosynthesis and deposition of cuticular wax on the leaves of EMS-mutagenized *Dianthus spiculifolius* plants in the M_2_ generation (Zhou et al., 2018). CER26 is involved in the elongation of the very long chain fatty acids of 30 carbons or more, and its mutation substantially decreased the amount of these very long chain fatty acids (Pascal et al., 2013). The mutation, however, did not affect the wax load on leaf or stem. In our study, we observed a significant difference in leaf cuticular wax content between the WT waxy and M_3_ glossy mutant lines. The wax load in the WT waxy was 3.5 ± 0.17 mg cm^-2^, whereas it was 1.0 ± 0.06 mg cm^-2^ in the glossy mutant lines (**Figure 3C**). Significant suppression of the four 4 critical wax biosynthesis genes (*AcCER1*, *AcMAH1*, *AcWSD1* and *AcCER26*) in our glossy mutant may have disrupted or slowed down the acyl reduction and decarbonylation pathways of wax biosynthesis, resulting in 3.5 times lower cuticular wax load in the glossy mutant than the WT waxy.

Our research findings indicated that within the wax biosynthesis pathway, *CER1* displayed the highest significant downregulation among the four downregulated genes. These results are consistent with the earlier study by Pu et al. (2013), which showed notable downregulation of *CER1* (4.13-fold) and mild downregulation of other wax biosynthesis-related genes, including *MAH1, WSD1, CAC3, FATB, ABCG28*, and *FAR* (ranging from 1.38 to 1.80-fold), in the glossy mutant of *Brassica napus*. Another study conducted by Liu et al. (2014) also revealed downregulation of four wax biosynthesis pathway genes, namely *LACS2, ECR3 (WAX2 isoform 1), ECR3 (Fatty acid hydroxylase* superfamily*), and ABCG12* (1.3 to 1.55-fold), with *LACS2* exhibiting the most substantial downregulation (5-fold) in glossy mutant of Welsh onion. The variation in downregulated genes and their expression levels in glossy mutants might be influenced by the specific genotype and the regulatory gene that was prabably mutated. The glossy mutant has potential applications in breeding. For instance, the male sterile parent could act as a reliable indicator for eliminating rogue plants in hybrid seed production, as well as in genetic studies focused on wax biosynthesis and export. Compared to the waxy-type, the glossy phenotype of onion has been associated with significantly less epicuticular wax and has been found resistant to thrips infestation (Damon et al., 2014). The quantity of epicuticular wax deposition is directly to insect resistance in various crops such as *Sorghum bicolor* (Starks and Weibel, 1981), *Triticum aestivum* (Lowe et al., 1985), *Brassica oleracea* var. *capitata* (Voorrips et al., 2008) and *Allium cepa* (Damon et al., 2014). Additionally, novel genes associated with cuticular wax biosynthesis potentially serve as valuable genetic resources for enhancing drought resistance in crops (Xue et al., 2017). Considering these factors, we anticipate that our glossy mutant phenotype could offer a potential advantage in onion breeding, contributing to the development of lines with increased resistance to biotic and abiotic stressors.

## Conclusion

The wax layer on the epidermal surface provides protection to terrestrial plants from UV radiation, water loss and gas exchange, and biotic stresses. In this study, we isolated a glossy mutant from the M_2_ population of γ-irradiated onion mutant lines in variety B. Super. The glossy trait is controlled by a recessive locus. The mutant accumulates wax poorly on the leaf surface, and we show that there is a concomitant downregulation of four structural genes in the wax biosynthesis pathway, namely *AcCER1*, *AcCER26*, *AcWSD1*, and *AcMAH1*, which potentially explains the reduction in wax accumulation. Single gene inheritance indicates that the mutation could be within a regulatory gene that controls these structural genes. Therefore, it would be worthwhile to map this regulatory locus using markers identified around this locus. Additionally, the glossy mutant can be a valuable addition to studies on both abiotic and biotic stress in plant breeding. We will conduct further investigation to determine the influence of cuticle thickness and the epicuticular waxes in conferring resistance to onion plants against various environmental stressors and pests.

## Data availability statement

All data are included in the article. RNA-seq raw data is available at the NCBI database (https://www.ncbi.nlm.nih.gov/bioproject/PRJNA943431).

## Author contributions

TM and SA conceived and designed the experiments. TM, SS, and YK conducted all the experiments. TM and SS drafted the manuscript. SA and VS carried out the data analysis and manuscript editing. SA supervised the entire work.
