# Supplementary material for: A glossy mutant in onion (Allium cepa L.) shows decreased expression of wax biosynthesis genes
Source: Front Plant Sci. 2023 Aug 23;14:1245308. doi: 10.3389/fpls.2023.1245308 (PMC10482397; doi:10.3389/fpls.2023.1245308)
Supplement: Supplementary file 1 [file DataSheet_1.docx]

**Supplementary Information**

**Supplement S1**

**A *glossy* mutant in onion (*Allium cepa* L.) shows decreased expression of wax biosynthesis genes**

Journal: Scientific Reports

Tushar K. Manape^1†^, Soumia P S^1†^, Yogesh P. Khade^1^, Viswanathan Satheesh^2^ & Sivalingam Anandhan^1^*

^1^ICAR-Directorate of Onion and Garlic Research, Rajgurunagar, Pune, Maharashtra, 410 505, India.

^2^Genome Informatics Facility, Office of Biotechnology, Iowa State University, Ames, Iowa, 50010, USA.

*Corresponding author: anandhans@gmail.com

**ORCID ID of the corresponding author: 0000-0002-1310-0807**

^†^ These authors contributed equally to this work.

**Supplementary Information S1**

**RNAseq sequence analysis and assembly**

RNA-seq of 6-weeks-old leaves was performed on two biological replicates each of wild-type (C1 and C2) and glossy mutant (G1 and G2) using an Illumina NovaSeq6000 platform with 2x150 bp chemistry. After stringent quality assessment and data filtering, a total of 104.33 million pair end reads, corresponding to 30.26 Gb of sequence data (Table S1), were generated. The high-quality reads all 4 samples were pooled and used to assemble 2,62,867 transcripts with a mean length of ~842 bp using Trinity de novo assembler (Table S2). These assembled transcripts were then further clustered to generate 69,169 validated unigenes with an average length of ~1,329 bp (Table S2). The N50 values of the transcripts and unigenes were 1,372 bp and 1,855 bp, respectively (Table S2).

**CDs prediction, functional annotation and classification:**

A total of 33,392 were predicted out of 69,169 pooled unigenes using TransDecoder v5.3.0. To identify sample-wise CDS from the pooled set, reads from each of the samples (C1, C2, G1, G2) were mapped to the final set of pooled CDS using bowtie (version) mapper. Statistics for sample-wise CDS are summarized in Table S3. Functional annotation of the CDS was carried out using DIAMOND program (BLASTX alignment mode) against the NCBI NR (non-redundant protein database). Of the total 33392 pooled CDS, 26398 CDS had hits, while 6994 CDS were not annotated. The majority of the blast hits were against *Asparagus officinalis*. Gene ontology (GO) analyses of the identified CDS for each of the 4 samples were carried out using Blast2GO program. A total of 6396, 7531, 6958 and 6783 CDS from the C1, C2, G1 and G2 samples, respectively, were categorized into 3 different GO categories (Table S4).

**Pathway Enrichment Analysis**

To identify the potential involvement of the predicted CDS in biological pathways, all the identified CDS for each of the 4 samples were mapped to reference canonical pathways in KEGG (Eudicots database). The identified CDS for all the 4 samples were found to be categorized into 23 KEGG pathways under five main categories: Metabolism, Genetic information processing, Environmental information processing, Cellular processes and Organismal systems. A total of 5890, 6029, 5768 and 5661 CDS were annotated in KEGG pathway analysis out of 19,955, 22,727, 19,735 and 18,592 annotated CDS of sample C1, C2, G1 and G2, respectively. The details of KEGG pathway classification of all the samples is summarized in the Table S5.

**Table S1:** High quality read statistics of 4 samples

| Description | C1 | C2 | G1 | G2 |
| --- | --- | --- | --- | --- |
| No. of PE Reads | 25,463,122 | 28,524,397 | 22,990,966 | 27,356,490 |
| Total Number of bases | 7,378,418,879 | 8,304,753,091 | 6,665,728,852 | 7,898,118,061 |
| Total data in Gb | 7.38 | 8.31 | 6.67 | 7.90 |

**Table S2:** Transcripts and unigens summary

| Description | Transcripts (pooled) | Unigenes (pooled) |
| --- | --- | --- |
| Total Number | 2,62,867 | 69,169 |
| Total length (bp) | 221,345,803 | 91,966,059 |
| N50 (bp) | 1,372 | 1,855 |
| Maximum length (bp) | 15,610 | 15,610 |
| Minimum length (bp) | 176 | 201 |
| Mean length (bp) | ~842 | ~1,329 |

**Table S3:** Sample-wise CDS statistics

|  | C1 | C2 | G1 | G2 |
| --- | --- | --- | --- | --- |
| No. of CDS | 19,955 | 22,272 | 19,753 | 18,592 |
| Total CDS length (bp) | 23,448,747 | 26,373,663 | 22,947,039 | 21,337,719 |
| Maximum CDS length (bp) | 14,970 | 14,970 | 10,827 | 10,827 |
| Minimum CDS length (bp) | 258 | 258 | 258 | 258 |
| Mean CDS length (bp) | ~1,175 | ~1,184 | ~1,161 | ~1,147 |

**Table S4:** GO category distribution of CDS for 4 samples

| Sample Name | Biological process | Cellular process | Molecular process | Total |
| --- | --- | --- | --- | --- |
| C1 | 2,388 | 1,132 | 2,876 | 6,396 |
| C2 | 2,464 | 2,116 | 2,951 | 7,531 |
| C3 | 2,266 | 1,962 | 2,730 | 6,958 |
| C4 | 2,207 | 1,918 | 2,658 | 6,783 |

**Table S5:** KEGG Pathway classification summary of CDS

| Pathways | # CDS Counts | | | |
| --- | --- | --- | --- | --- |
|  | C1 | C2 | G1 | G2 |
| **Metabolism** | | | | |
| Carbohydrate metabolism | 529 | 556 | 534 | 523 |
| Energy metabolism | 337 | 331 | 331 | 336 |
| Lipid metabolism | 306 | 310 | 285 | 287 |
| Nucleotide metabolism | 133 | 134 | 125 | 127 |
| Amino acid metabolism | 336 | 334 | 318 | 319 |
| Metabolism of other amino acids | 162 | 165 | 158 | 159 |
| Glycan biosynthesis and metabolism | 132 | 138 | 122 | 115 |
| Metabolism of cofactors and vitamins | 279 | 289 | 269 | 255 |
| Metabolism of terpenoids and polyketides | 114 | 118 | 108 | 104 |
| Biosynthesis of other secondary metabolites | 114 | 122 | 106 | 111 |
| Xenobiotics biodegradation and metabolism | 84 | 82 | 80 | 79 |
| **Genetic Information Processing** | | | | |
| Transcription | 289 | 294 | 291 | 282 |
| Translation | 602 | 615 | 600 | 578 |
| Folding, sorting and degradation | 517 | 533 | 518 | 508 |
| Replication and repair | 110 | 124 | 112 | 103 |
| **Environmental Information Processing** | | | | |
| Membrane transport | 32 | 34 | 31 | 26 |
| Signal transduction | 619 | 639 | 600 | 601 |
| **Cellular Processes** | | | | |
| Transport and catabolism | 432 | 441 | 427 | 416 |
| Cell growth and death | 272 | 282 | 265 | 263 |
| Cellular community - eukaryotes | 90 | 90 | 90 | 83 |
| Cellular community - prokaryotes | 48 | 53 | 52 | 48 |
| **Organismal Systems** | | | | |
| Environmental adaptation | 306 | 299 | 299 | 298 |

**Table S6** Primers and PCR conditions used for semi-quantitative PCR

| **Primer Name** | **5’ to 3’ sequence** | **Amplicon size** | **PCR conditions** |
| --- | --- | --- | --- |
| *AcMAH1-*F | CTGTGGTTGGGGAGAGGATA | 211 bp | 1 cycle of 94°C for 3 min; 26 cycles of 94°C for 30 s, 58°C for 30 s, 72°C for 30 s and  1 cycle of 72°C for 10 min |
| *AcMAH1-*R | GTCGTCTTCAACTGCACGAA |  |  |
| *AcCER1-*F | CTCGCTATCACTCCCACCAT | 202 bp | 1 cycle of 94°C for 3 min; 26 cycles of 94°C for 30 s, 58°C for 30 s, 72°C for 30 s and  1 cycle of 72°C for 10 min |
| *AcCER1-*R | TTGCAGTGACCCATGTTGTT |  |  |
| *AcCER26-*F | CGGATTCCGTGAAATCCTTA | 242 bp | 1 cycle of 94°C for 3 min; 26 cycles of 94°C for 45 sec, 56°C for 45 sec, 72°C for 1 min and 1 cycle of 72°C for 10 min |
| *AcCER26-*R | CATGTACCCACCCACCCTAC |  |  |
| *AcWSD1-*F | CAGTACCTCAACAGCGACCA | 225 bp | 1 cycle of 94°C for 3 min; 28 cycles of 94°C for 30 s, 58°C for 30 s, 72°C for 30 s and  1 cycle of 72°C for 10 min |
| *AcWSD1-*R | CCCTGAGGGAAATGTTGGTA |  |  |
| *AcEF1α-*F | TAAGGCAGAGCGTGAAAGAGG | 218 bp | 1 cycle of 94°C for 3 min; 24 cycles of 94°C for 30 s, 56°C for 30 s, 72°C for 30 s and  1 cycle of 72°C for 10 min |
| *AcEF1α-*R | GCTCACGGGTCTGACCATC |  |  |


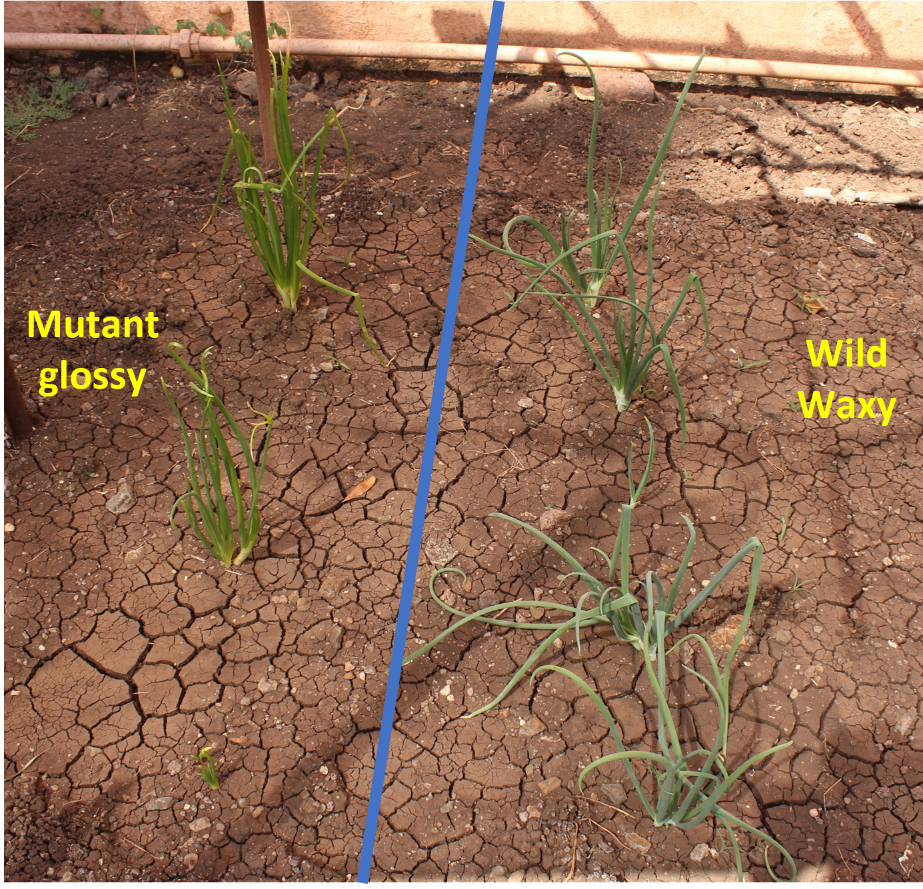


**Fig. S1 Phenotype of WT and glossy mutant**

Comparative field photographs of glossy phenotypes observed in the field grown M_2_ population of **γ-**irradiation treated onion mutant line (B. Super-IR-300-53). Right side panel shows WT waxy phenotypes.


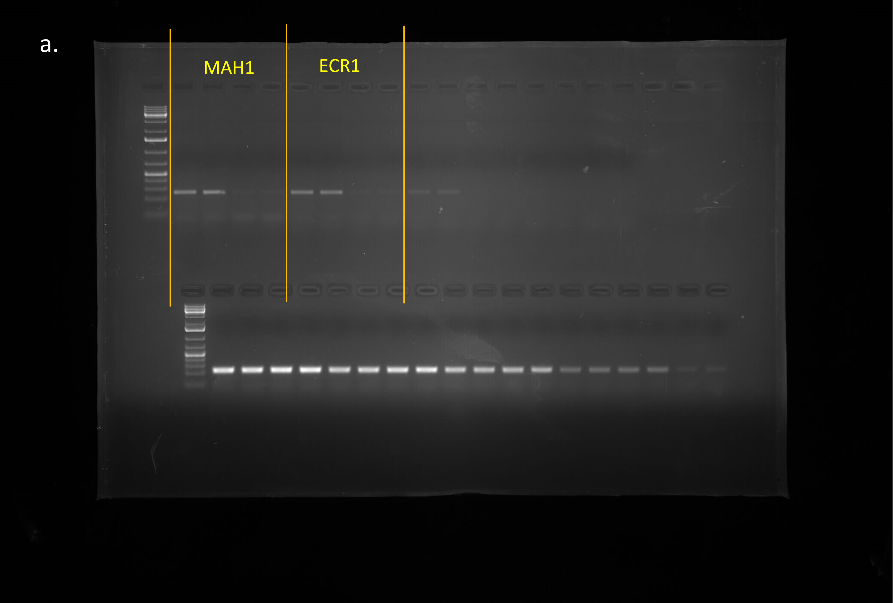


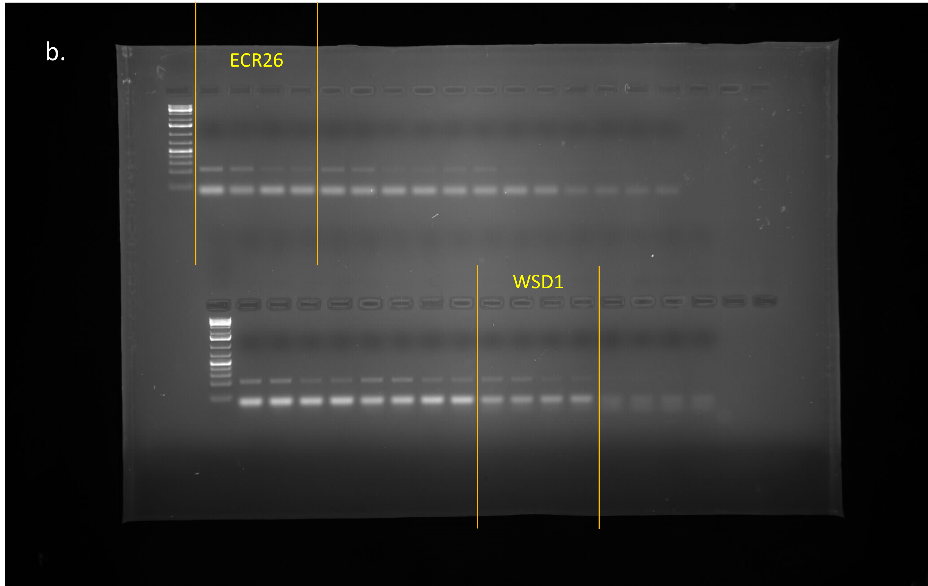


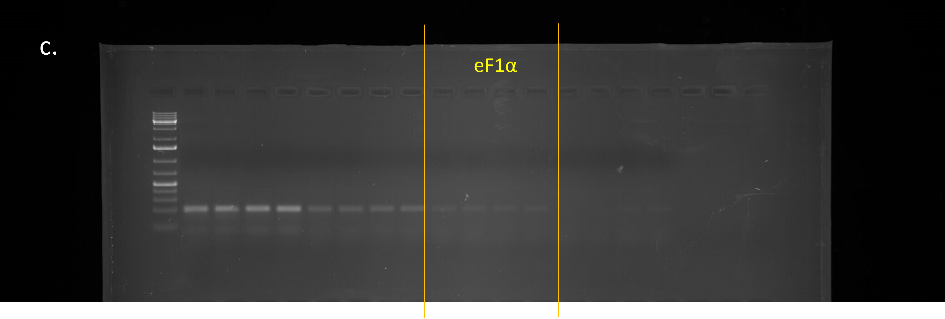


**Fig. S2 Original gel images used for semi-quantitative PCR analysis**

Gel images of PCR amplified products with a. MAH1 and ECR1, b. ECR26 & WSD1, c. eF1α gene specific primer sets. These gel images were converted into 8-bit scale using ImageJ analyzer tool for quantitative estimation of gene expression analysis. PCR amplified products within marked area were used for differential gene expression analyses.
